# Proposed research classification criteria for Lyme disease in infection associated chronic illness studies

**DOI:** 10.3389/fmed.2025.1519163

**Published:** 2025-02-25

**Authors:** Brian A. Fallon, Mara Kuvaldina, Nevena Zubcevik, Roberta DeBiasi, Sarah B. Mulkey, Charles Chiu, Felicia Chow, Kristopher Paolino, Randy Lai, David Putrino, Amy Proal, Martina Pavlicova, John Aucott

**Affiliations:** ^1^Lyme & Tick-Borne Diseases Research Center at Columbia University Irving Medical Center, New York, NY, United States; ^2^Department of Psychiatry, Columbia University, New York, NY, United States; ^3^New York State Psychiatric Institute, New York, NY, United States; ^4^Department of Physical Medicine and Rehabilitation, Harvard Medical School, Spaulding Rehabilitation Hospital, Boston, MA, United States; ^5^Children’s National Hospital and Research Institute, Washington, DC, United States; ^6^The George Washington University School of Medicine, Washington, DC, United States; ^7^Clinical Microbiology Laboratory, School of Medicine, University of California, San Francisco, San Francisco, CA, United States; ^8^UCSF-Abbott Viral Diagnostics and Discovery Center (VDDC), University of California, San Francisco, San Francisco, CA, United States; ^9^Weill Institute for Neurosciences, School of Medicine, University of California, San Francisco, San Francisco, CA, United States; ^10^Institute for Global Health and Translational Sciences, Upstate Medical University, Syracuse, NY, United States; ^11^Department of Rehabilitation and Human Performance, Icahn School of Medicine at Mount Sinai, New York, NY, United States; ^12^PolyBio Research Foundation, Medford, MA, United States; ^13^Mailman School of Public Health, Columbia University Irving Medical Center, New York, NY, United States; ^14^Lyme Disease Research Center, Baltimore, MD, United States; ^15^Division of Rheumatology, Johns Hopkins University School of Medicine, Baltimore, MD, United States

**Keywords:** infection associated chronic illness, post-treatment Lyme disease, research criteria, clinical trials, inclusion/exclusion criteria, chronic Lyme disease, clinical trials network

## Abstract

**Background:**

Research on patients with persistent symptoms despite prior treatment for Lyme disease can be challenging to interpret given the diversity of criteria selected to characterize Lyme disease and to define the syndrome of those with persistent symptoms. Because most research studies only include patients with well-documented prior Lyme disease, the generalizability of the study results is limited, excluding the larger group of patients often seen in community practice who do not meet these stringent enrollment criteria. Researchers at the Lyme and other Tick-borne Diseases Clinical Trials Network (LTD-CTN) recognized early on that a research classification system was needed to facilitate the design of studies that are more inclusive. This paper presents a proposed research classification system.

**Methods:**

Criteria used in published clinical research on previously treated Lyme disease were reviewed. Clinical expertise was provided by principal investigators in the LTD-CTN. Further input was obtained from a diverse panel of stakeholders in the field, including clinicians, academic researchers, and patient advocates. This classification system was developed based on feedback collected from all these sources.

**Results:**

The new research classification system proposes criteria for Lyme disease at different levels of diagnostic certainty: well-defined, probable, possible, and uncertain. Criteria for ascertainment for each classification level and additional factors to be considered in patient selection for research are described.

**Conclusion:**

The proposed research classification system should improve the quality and generalizability of clinical research by providing clear case definitions for enrollment of a more diverse group of patients with sequelae from Lyme disease.

## 1 Introduction

Lyme disease, caused in the United States primarily by the microbe *Borrelia burgdorferi*, is the most common vector borne disease, with nearly 500,000 new cases diagnosed each year (1). While many patients who are treated early in infection recover fully, approximately 10–20% of patients experience relapsing or persistent symptoms, such as fatigue, musculoskeletal pain, neuropathies, mood disorders, and/or cognitive problems, associated with functional impairment and/or reduced quality of life (2–7). Terms used to describe individuals in this group include post-treatment Lyme disease syndrome (PTLDS), post-treatment Lyme disease (PTLD), chronic Lyme disease, persistent Lyme disease, Long Lyme, and most recently Lyme Infection-Associated Chronic Illness (Lyme IACI) (6, 8–10). PTLDS, a more narrowly defined term, has been used in research publications (4–6). The broader more inclusive term of Infection Associated Chronic Illness (IACI) has been employed by subject experts in recent meetings in the United States conducted by Department of Health and Human Services (11) and by the National Academies of Science, Engineering, and Medicine (NASEM) (10), triggered by recognition that many serious infections (e.g., SARS CoV-2, *Borrelia burgdorferi*) may lead to chronic symptoms and that there may be common pathophysiologic mechanisms and treatments across IACI. It has been estimated that there are 1–2 million individuals in the United States living with persistent Lyme disease symptoms (12). Given the magnitude of this population with chronic symptoms, a better understanding of mechanisms of disease and treatment strategies is a public health priority (10).

In 2020, a clinical trials network for the study of Lyme and other tick-borne diseases (LTD-CTN) was established in the United States to facilitate clinical trials capable of identifying more effective treatments for patients with acute and persistent manifestations of Lyme and other tick-borne diseases (10). The coordinating center of the LTD-CTN, located at Columbia University Irving Medical Center in New York City, serves as the hub of a team of investigators from academic sites throughout the United States. The group of patients with Lyme IACI is a primary focus of the LTD-CTN, as there remains considerable uncertainty and controversy about how to treat these patients. The primary goal therefore of the LTD-CTN is to address the lack of FDA-approved treatments for patients with persistent symptoms by conducting clinical trials of potentially effective therapies. Research at the LTD-CTN aims to target the different mechanisms that may account for or contribute to persistent symptoms, such as persistent infection, autoimmunity, inflammation, dysregulated central or peripheral nervous system, and dysbiosis (13).

An initial challenge for the LTD-CTN was to define the patient sample of those with persistent symptoms, as the symptom presentations are diverse and the quality of documentation of prior Lyme disease varies. Indeed, prior research on these patients is at times challenging to interpret given the lack of clarity on the criteria selected to characterize Lyme disease and to define the syndrome of those with persistent symptoms. Further confounding is that some published studies clearly state the details of the diagnostic inclusion and exclusion criteria and ascertainment methods, while other studies do not.

In 2006 the Infectious Diseases Society of America published a proposed case definition for Post-treatment Lyme disease syndrome (PTLDS) with clear inclusion and exclusion criteria (6); this was later operationalized to facilitate research (4). The criteria for PTLDS required documentation that the initial Lyme disease diagnosis met the surveillance case definition established by the Council of State and Territorial Epidemiologists (CSTE) (and used by the Centers for Disease Control and Prevention (CDC)), a symptom onset within the 6 month period following diagnosis and treatment of Lyme disease, and functional impairment; symptom categories included fatigue, widespread musculoskeletal pain, and cognitive complaints. There were many exclusionary criteria, including ruling out other causes of the typical symptoms. Also excluded were individuals with antibiotic-refractory Lyme arthritis, residual neurologic impairment from facial palsy, or cognitive problems confirmed with neuropsychological testing.

While some studies of PTLDS since 2006 relied on this case definition, other studies included a broader range of patients who met only some of the criteria. Examples of these modifications include studies that enrolled patients with cognitive problems evident on neurocognitive testing, as this is a common feature of patients with persistent symptoms, or studies that included those with typical symptoms who had positive serologic tests but without a documented history of prior objective signs of Lyme disease (e.g., erythema migrans, facial palsy). Confirmation of eligibility in some studies required physical documentation of medical history, while in other studies the patient’s self-report was sufficient for enrollment.

The use of stringent inclusion criteria in studies of persistently symptomatic previously treated Lyme disease that limits enrollment to individuals with well-documented prior Lyme disease meeting the surveillance case definition increases the homogeneity of the study population; this is of particular importance when the goals of the study are to develop new diagnostic assays, to test new antibiotic treatments, to identify biomarkers of treatment response, and/or to clarify pathogenesis. However, such diagnostic rigor has the negative impact of limiting the generalizability of the study results, excluding the much larger group of patients who have symptoms consistent with PTLDS but for whom the diagnosis cannot be confirmed. Lack of confirmation of the initial diagnosis may be due to many factors, most common of which are difficulty retrieving older medical records or absence of objective clinical signs or tests. These issues raise the question of whether this larger group of less clearly defined patients can be studied.

Similar challenges relating to broad vs. narrow classification criteria have been faced by other investigators in the infection-associated chronic disease field. A recent example is Long COVID (aka Post-Acute Sequelae of SARS-CoV-2 infection or PASC). In 2024, the National Academies of Sciences, Engineering and Medicine convened a panel of experts to define Long COVID based on their expertise, the medical literature, and stakeholder and patient input. The consensus definition stated that “Long COVID (LC) is an infection-associated chronic condition (IACC) that occurs after SARS-CoV-2 infection and that is present for at least 3 months as a continuous, relapsing and remitting, or progressive disease state that affects one or more organ systems” (14). Notable is that this definition emphasizes inclusivity as it does not require laboratory confirmation or other proof of the initial infection, thereby including both confirmed and suspected prior SARS CoV-2 infection.

Reviews of clinical trials of previously treated Lyme disease indicate that studies may exclude over 90% of those who are screened (3, 15). Stringent inclusion criteria lead to study results that may only apply to a narrow spectrum of the patient community, leaving front-line clinicians in the dark as to how to help the larger group of patients with persistent symptoms. The impact of specific inclusion and exclusion criteria on enrollment was demonstrated recently in a study of a patient registry (“MyLymeData”) of over 17,000 individuals with a history of clinician diagnosed Lyme disease (10). The report indicated that when patients were required to have a history of a rash or a positive Western blot, 35% of the sample were eliminated. When patients were additionally required to have characteristic symptoms and no prior diagnosis of Myalgic Encephalitis/Chronic Fatigue Syndrome (ME/CFS) or fibromyalgia, only 39% of the original sample remained. This report highlights the challenges in designing research studies, given the need to balance the benefits of stringent enrollment criteria for targeted aims against the benefits of broadening enrollment criteria to enhance generalizability and study feasibility (10, 16).

Early in the history of the LTD-CTN, it became clear to the investigators that the 2006 IDSA case definition was too restrictive given the large numbers of patients excluded from clinical trials (6). A research classification system was needed to enable inclusion of those patients who have a history of both well-documented and those with less-well documented Lyme disease. Such a research classification system would reflect the diversity of patients who are treated for Lyme disease in the community setting, including individuals with delayed treatment due to initial misdiagnosis (17), and would expand the generalizability of study results.

This paper proposes a new research classification system of Lyme disease describing different levels of diagnostic certainty with the goal that it may assist in the design of more inclusive research studies, while still allowing investigators to choose the level of enrollment specificity they need to address their study goals. This proposed system is *for research only* and not for decision making in the clinical setting. In addition to application to studies of Lyme IACI, this classification system can be helpful for studies of acute Lyme disease, particularly if one wishes to examine the question of treatment efficacy among individuals who do not meet all of the standard clinical and serologic criteria for Lyme disease (18). This proposed research classification system should improve the quality of clinical research by providing clear case definitions for study design and enrollment and by enhancing ease of entry into research studies. Broader enrollment criteria will deepen our understanding of the larger group of patients with sequelae from Lyme disease.

## 2 Methods

The development of this research classification system was led by clinical investigators with extensive experience in diagnosing Lyme disease and its sequelae during a series of consensus meetings. We reviewed published clinical trials and retrospective treatment studies of individuals with persistent symptoms after Lyme disease to assess the criteria used for subject inclusion and exclusion. Investigators from the CTN met biweekly for several months to develop and refine the current proposed classification scheme. Further input was obtained from a diverse panel of stakeholders in the field, including community and academic clinicians, researchers from both academic and public health centers, and patient advocates.

## 3 Results

Table 1 lists some of the clinical and historical criteria selected in prior research studies to enroll participants with previously treated Lyme Disease. The new research classification system (Table 2) proposes criteria for Lyme disease at 4 different levels of diagnostic certainty: well-defined, probable, possible, and uncertain. Standardized methods of ascertainment are described to ensure criteria are met.

**Table 1:** Enrollment criteria considered in prior studies of previously treated Lyme disease.

Typical signs of Lyme disease by history (i.e., not symptoms alone)
Typical persistent symptoms of previously treated Lyme disease
Severity level of symptoms and/or of functional impairment
Symptom onset coincident or within a window of time after diagnosis and treatment
Persistent symptoms are either continuous or relapsing/remitting
Specific maximum or minimum duration of illness prior to enrollment
Laboratory test confirmation (e.g., standard reference criteria &/or “in-house” criteria)
History of exposure to a Lyme endemic area
Prior antibiotic therapy for Lyme disease (e.g., a minimum or maximum amount)
Exclusion of pre-existing confounding disorders/diseases/infections
Ascertainment methods: self-report; clinician records; pharmacy records; and/or lab test records

**Table 2:** Proposed research classification criteria for Lyme disease.

Group 1. Well-defined Lyme disease
Early Lyme disease
Erythema migrans rash (EM) EM 1: diagnosed by health care provider (HCP) (either in person or telemedicine) and occurring after exposure to a suspected Lyme endemic area or high incidence state EM 1A: Method of ascertainment (MOA): self-report and medical record documentation of an EM rash EM 1B: MOA: self-report of HCP diagnosed EM and either: photo of EM or Class 1 lab test confirmation
OR
Disseminated “Objective” manifestations
Clinical history includes at least one of the following symptoms/signs, which are not better accounted for by another cause (MOA: medical records and/or self-report). Neurologic: lymphocytic meningitis; encephalitis; encephalomyelitis, cranial neuritis (especially facial palsy); radiculoneuropathy. Other neurologic signs (with objective measures): encephalopathy, polyneuropathy Carditis: Acute onset 2^nd^ or 3rd degree atrioventricular conduction defects; myocarditis; pericarditis Arthritis: Recurrent joint swelling in one or more joints Dermatologic: Multiple EM rashes or Acrodermatitis chronica atrophicans
AND
2. Lab test Confirmation (requires at least one of the Class 1 lab tests) (MOA: self-report & documentation)

Group 2. Probable Lyme Disease.
2A. Chronic Multisystem Symptoms attributed to Lyme disease (insufficient to meet Group 1), not better explained by another diagnosis, and evidence of a positive Class 1 lab test or a highly suggestive IgG Western blot (WB) or Immunoblot (IB) (MOA: self-report with lab documentation) MOA: Class 1 lab test confirmation (excluding IgM WB) MOA: Highly suggestive IgG WB or IB (e.g., at least 4 of 10 IgG Bands)
OR
2B. EM rash after exposure to a suspected Lyme endemic area or a high incidence state but not previously diagnosed as EM by a health care provider and no photo or Class 1 lab test confirmation is available. MOA: self-report and medical record confirming a prior rash consistent with EM but not recognized as an EM at that time (e.g., misdiagnosis as cellulitis or spider bite) MOA: self-report only but clinical history consistent with EM (i.e., no medical record or photo or positive Class 1 test)
OR
2C. Viral like illness (not better explained by another cause) after exposure to a suspected Lyme endemic area or a high incidence state with subsequent lab tests showing either: (a) positive Class 1 lab test; or (b) Enzyme Immunoassay (EIA) (indeterminate or positive) & positive IgM WB within 6 months of the viral illness (specify if within 4 weeks or between 4 weeks and 6 months) MOA: medical records, lab test and self-report MOA: lab test and self-report

Group 3. Possible Lyme Disease.
Individuals not meeting criteria for Groups 1 or 2 but who have multi-system symptoms not better explained by another diagnosis with evidence of possible prior exposure to *Bb* based on a Lyme disease test. (MOA: self-report and lab documentation)
1. History of suspected exposure to a Lyme endemic area or a high incidence state
AND
2. History of signs or symptoms consistent with Lyme disease (with or without prior health care provider recognition)
AND
3. A positive or highly suggestive past Lyme assay not sufficient to meet Group 1 or 2 above

Group 4. Uncertain.
Those who do not meet any of the above criteria but for whom a provider has clinically diagnosed the individual with suspected Lyme disease (MOA: self-report).
Class 1 laboratory tests (performed in a CLIA-certified lab)
Standard two-tier tests (STTT): a positive or equivocal enzyme immunoassay (EIA) or immunofluorescence assay (IFA) as the first step and western blotting (WB) as the second step. ○ Positive IgM Western blot (WB)/Immunoblot (IB) is sufficient when using the time frame guidelines indicated for Group 1 (within 4 weeks of illness onset) or Group 2 (within 6 months of illness onset). Requires at least 2 of 3 bands (Osp C, 39, 41 kDa). ○ Positive IgG WB/IB is sufficient at any time. Requires at least 5 of 10 bands (18, Osp C, 28, 30, 39, 41, 45, 58, 66, 93 kDa). Modified two-tier test (MTTT): positive or equivocal first-tier screen, followed by a different positive or equivocal confirmatory FDA-cleared EIA (13) Any FDA-cleared test for Lyme disease: 510(k) Premarket Notification link: https://www.accessdata.fda.gov/scripts/cdrh/cfdocs/cfPMN/pmn.cfm. Search link using Lyme, Borrelia, or name of manufacturer or device. Positive single-tier IgG Western blot/Immunoblot (at least 5 IgG bands) Positive *Bb* specific EIA (for example, C6 or another VlsE-based Peptide EIA) Detection of *Borrelia burgdorferi or B. mayonii* by PCR Isolation of *Borrelia burgdorferi* or *B. mayonii* in culture

These proposed criteria are meant to assist researchers as they design research studies on Lyme disease to allow for greater inclusiveness and generalizability. The selection of criteria and methods of ascertainment (MOA) depend on the study goals.

### 3.1 Notes for Table 2 research classification criteria

#### 3.1.1 Exposure

Individuals meeting criteria for groups 1–4 have had exposure to suspected Lyme endemic areas or high incidence states, either through residence or travel. In the U.S., a Lyme endemic county was defined by the Council of State and Territorial Epidemiologists (CSTE) as an area that has had at least two confirmed cases of Lyme disease acquired in the county or an established population of infected *Ixodes* ticks. In 2017, the CSTE, in recognition of the limitations of the definition of “endemic,” modified the case definition to focus more on “high incidence” vs. “low incidence” jurisdictions (19, 20). As the geographic expansion of infected ticks continues, additional states will be included in the “high incidence” category. “High Incidence” jurisdictions in the U.S. in 2022 (≥10 confirmed cases of Lyme disease/100,000 for a period of 3 years) included: Connecticut, Delaware, Maine, Maryland, Massachusetts, Minnesota, New Hampshire, New Jersey, New York, Pennsylvania, Rhode Island, Vermont, Virginia, West Virginia, Wisconsin, and the District of Columbia (21). See CDC annual reports for further updates.

## 4 Discussion

There are four main foreseeable benefits from implementation of this research classification system. First, it will facilitate the design of future studies that include participants with less well-defined Lyme disease. This enhances generalizability, expands knowledge about this understudied population, and increases the speed of completion of clinical trials as a larger population of subjects will be eligible for study enrollment. Second, use of this research classification system will improve the ease and precision with which clinical researchers can characterize their sample in publications and presentations. Third, this system, if used widely, will enable more direct and informed comparison of treatment and biomarker results across research studies. Fourth, this system will enable subgroup comparisons within studies, as subgroups may differ in their pathophysiology and in their treatment response.

Along with these benefits, there is a major risk to enhancing heterogeneity in a clinical trial without clearly defining subpopulations in advance, as heterogeneity may mask treatment effects that are actually present within one classification level but not another. Using this classification scheme, decisions can be made as to the appropriate proportion of subjects to be enrolled from each classification level to ensure achievement of the specific study goals; randomization to treatment can be stratified within each classification level. As in all studies of Lyme IACI, the criteria for enrollment should exclude individuals for whom there is another better explanation for the persistent symptoms.

Clinical trials of patients with persistent symptoms despite prior treatment for Lyme disease are costly, partly because recruitment rates are slow due to narrow criteria for entry and because it is often challenging for patients to obtain confirmatory documentation. As noted previously, 9 of 10 screened participants are excluded from studies of previously treated Lyme disease when the original epidemiologic surveillance case definition criteria have been used to confirm prior Lyme disease. Improving recruitment rates will make clinical trials more efficient and productive.

There are ethical, social, scientific, and pragmatic reasons to design trials that have more inclusive enrollment criteria. A look at clinical trials of Lyme disease in the United States indicates that less than 5% of included subjects are from racial or ethnic minority groups (3, 15, 22). Classification criteria for Group 1, for example, which represents the standard criteria for publication in highly ranked journals, requires medical documentation of the rash or of laboratory tests of early Lyme disease; this presumes easy access to health care and the ability to cover the expense of evaluation and testing. People with financial or social disadvantages may not be able to access health care quickly, making it unlikely that these individuals would be evaluated at the initial signs of infection; this group would therefore be excluded from a clinical trial if a health care provider’s documentation of the early signs of Lyme disease was required for study entry. Further challenging ease of study entry is that the most common manifestation of Lyme disease, the erythema migrans rash, may be difficult to recognize in individuals with darker skin; studies that rely on documentation of this early sign of Lyme disease would have less racial and ethnic diversity (17). Broadening the inclusion criteria, as outlined in the proposed research classification system, will consequently improve the ability of minoritized patients to participate in trials, thus reducing inequity due to access disparities to quality healthcare.

Limitations of this classification system include the fact that these criteria were delineated for use in studies in North America; the criteria may need modifications for studies in Europe given their more diverse genospecies and somewhat different clinical features (e.g., *Borrelial lymphocytoma*) and different laboratory criteria for confirmation (23, 24). Second, laboratory tests for Lyme disease are imperfect with known limitations in sensitivity and specificity at different stages of illness (25). The focus on the Western blot over the last two decades has been problematic as the original two-tier assays relied upon antigens derived from whole-cell cultured *B. burgdorferi*; these assays did not include antigens expressed only *in vivo* and included antigens which may bind non-Borrelia antibodies due to epitope cross-reactivity (25). Testing for Lyme disease is rapidly evolving with newer tests providing better sensitivity and specificity (26). The Western blot is included in our classification system because potential study participants with persistent symptoms spanning a decade or more will present with tests conducted using both older and/or more novel technologies. For the purposes of research studies, our classification system includes suggestions for laboratory testing for each of the classification groups. To keep the Class 1 assay list at the end of Table 2 current, a link is provided at the end of the Classification table to assist researchers who wish to identify all Lyme disease assays that have been FDA cleared, including those newly approved.

Ideally, a classification system for patients with acute Lyme disease or persistent sequelae of Lyme disease would utilize a more accurate and potentially quantitative test to enhance the confidence of diagnosis. This is especially important for the study of Lyme IACI, as there is considerable overlap in symptoms among all infection-associated chronic illnesses and conditions (e.g., Long COVID and ME/CFS). Laboratory tests that use a panel of biomarkers for differential diagnosis of early Lyme disease and Lyme IACI are in development. These include biomarkers based on multiplexed tickborne pathogen testing (27), proteomics and metabolomics (28), and host RNA gene expression (29, 30).

We conclude by emphasizing that this classification system is for research purposes, not for clinical application. Clinical decision-making regarding diagnostic and treatment decisions for patients with persistent symptoms is often challenging, as the symptom patterns although compatible with the diagnosis of Lyme disease are not specific, and the differential diagnosis is broad. Each patient needs to be evaluated as an individual, with attention to the diversity of inter-related factors that may contribute to the persistent symptoms and to the factors that may lead to false negative or false positive test results. In planning treatment strategies, the clinician needs to attend to the multiple possible mechanisms that may drive persistent symptoms, such as ongoing immune activation triggered by persistent infection or residual borrelia components, a dysregulated immune response (e.g., autoimmunity, inflammation), abnormally activated central nervous system networks, genetic vulnerability, environmental stressors, or the presence of other disorders or infections that may better explain the clinical picture (13). Given the diversity of individual clinical and immunologic responses to infection with *Borrelia burgdorferi*, some patients with Lyme disease will not meet the criteria for Groups 1–3.

## 5 Conclusion

The proposed research classification system should improve the quality of clinical research by providing standardized case definitions for enrollment. This will promote and support research that includes the larger group of patients with sequelae from Lyme disease who until recently have been largely excluded from research studies, while still maintaining the scientific rigor required to draw clinically relevant conclusions.

## Data Availability

The original contributions presented in the study are included in the article/supplementary material, further inquiries can be directed to the corresponding author.
